# COVID-19 in cancer patients: risk, clinical features, and management

**DOI:** 10.20892/j.issn.2095-3941.2020.0289

**Published:** 2020-08-15

**Authors:** Cuiwei Liu, Yanxia Zhao, Derick Okwan-Duodu, Reva Basho, Xiaojiang Cui

**Affiliations:** ^1^Cancer Center, Union Hospital, Tongji Medical College, Huazhong University of Science and Technology, Wuhan 430022, China; ^2^Department of Biomedical Sciences, Cedars-Sinai Medical Center, Los Angeles, CA 90048, USA; Derick Okwan-Duodu; ^3^Department of Pathology and Laboratory Medicine, Cedars-Sinai Medical Center, Los Angeles, CA 90048, USA; ^4^Department of Medicine, Samuel Oschin Comprehensive Cancer Institute, Cedars-Sinai Medical Center, Los Angeles, CA 90048, USA; ^5^Department of Surgery, Samuel Oschin Comprehensive Cancer Institute, Cedars-Sinai Medical Center, Los Angeles, CA 90048, USA

**Keywords:** Anti-viral therapy, anti-cancer treatment, COVID-19, immunotherapy, inflammation, SARS-CoV-2

## Abstract

A novel coronavirus known as severe acute respiratory syndrome coronavirus 2 (SARS-CoV-2) has spread across the world, prompting the World Health Organization to declare the coronavirus disease of 2019 (COVID-19) a public health emergency of international concern. Cancer patients are regarded as a highly vulnerable population to SARS-CoV-2 infection and development of more severe COVID-19 symptoms, which is possibly due to the systemic immunosuppressive state caused directly by tumor growth and indirectly by effects of anticancer treatment. Currently, much effort has been directed toward studying the pathogenesis and treatment of COVID-19, but the risk profiles, prognoses, and treatment outcomes in cancer patients remain unclear. Based on the current literature, we summarize the risk profiles, clinical and biochemical characteristics, and therapy outcomes of COVID-19 infections in cancer patients. The challenges in the clinical care of cancer patients with COVID-19 are discussed. The goal of this review is to stimulate research to better understand the biological impact and prognoses of COVID-19 infections in cancer patients, thus facilitating improvement of the clinical management of these patients.

## Introduction

A new coronavirus known as SARS-CoV-2 has spread throughout the world and caused the COVID-19 pandemic^1^. The symptomatic severity of COVID-19 infection seems to vary with age and the presence of comorbidities^2,3^. Older patients with chronic underlying diseases, such as cancers, are most vulnerable^2,4^.

An early study collected and analyzed 2007 COVID-19 cases from 575 hospitals in 31 provincial administrative regions in China^1^. Among these patients, 18 had a history of cancer, which may reflect a higher incidence of cancer than in the overall Chinese population (0.9% *vs.* 0.29%)^1^. This suggests that cancer patients might be more susceptible to SARS-CoV-2 infection. Although the number of COVID-19 cancer patients in this cohort was small, the study reported a higher incidence of acute complications in these patients compared with COVID-19 patients without cancer^1,5–7^. This finding highlights the need to evaluate clinical outcomes of COVID-19 infection and treatment in cancer patients. This review focuses on the clinical and biological features of COVID-19 infection in cancer patients, to stimulate research into strategies for improving the management of cancer patients with COVID-19.

## Risk of SARS-CoV-2 infection in cancer patients

In the COVID-19 pandemic, cancer patients are regarded as a highly vulnerable group due to weakened immune systems caused by both tumor growth and anti-cancer treatment^8–11^. Furthermore, given the confirmed nosocomial transmission of SARS-CoV-2 among patients in healthcare units^2^, cancer patients may be more prone to getting infected by SARS-CoV-2 due to contact with COVID-19 patients or virus-contaminated areas, because they need to regularly visit hospitals for anticancer therapy. In a cohort study of 105 cancer patients with COVID-19, Dai et al. reported that lung cancer was the most frequent cancer histology in infected patients (20.95%), followed by gastrointestinal cancer (12.38%), and breast cancer (10.48%)^6^. Another cohort study of 28 COVID-19 cancer patients reported that patients with Stage IV disease accounted for a higher percentage of infected patients (35.7%), suggesting that later stage cancer patients may be more susceptible to SARS-CoV-2^5^.

In a nationwide cohort study in China, Liang et al. found that the patients with cancer not only had a higher risk of SARS-CoV-2 infection, but also displayed an increased risk of severe clinical events (admission to the intensive care unit, need for invasive ventilation, or death) than those without cancer^1^. In this study, 39% of cancer patients with COVID-19 developed severe symptoms, compared with only 8% of non-cancer COVID-19 patients^1^. In a multi-center retrospective study of 105 cancer patients with COVID-19, it was found that patients with hematological malignancies and metastatic solid tumors had a relatively high risk of severe symptoms of 66.67 and 34.29%, respectively^6^. Another retrospective study reported that the overall fatality among 218 COVID-19 cancer patients from a single medical center in New York City exceeded 25%, which was 2-3-fold greater than their age-adjusted non-cancer counterparts^12^. Data from the COVID-19 and Cancer Consortium (CCC19) cohort study, which included 1,018 patients, also reported that mortality and severe illness in COVID-19 cancer patients were significantly higher than the general population^13^.

Notably, a retrospective study of 28 COVID-19 cancer patients found that anti-cancer treatment within 14 days before COVID-19 diagnosis was more frequently associated with severe clinical events due to SARS-CoV-2 infection^5^. Patients who underwent chemotherapy or surgery in the prior month had a significantly higher risk (75%) of clinically severe events than those who did not receive chemotherapy or surgery (43%)^1^. Similarly, another study of 205 COVID-19 cancer patients showed that receiving chemotherapy within 4 weeks before symptom onset and male sex were risk factors for death^14^. Furthermore, a multi-center study reported that COVID-19 cancer patients who received immunotherapy had higher percentages of severe symptoms (66.67%) and death (33.33%). In contrast, patients who received radiotherapy did not show a significant increase in the incidence of severe events^6^. Of note, thoracic cancer patients who received tyrosine kinase inhibitors had a decreased risk of hospitalization^15^.

Cancer survivors infected with SARS-CoV-2 also developed increased severity of COVID-19 symptoms compared to COVID-19 patients without a history of cancer^1^, suggesting that the immune surveillance mechanisms may not have fully recovered in patients with a history of cancer, thus resulting in a weakened defense against COVID-19 disease progression. Apart from disease and treatment-related factors, older age of many cancer patients is an additional risk factor for severe COVID-19 disease^1^. In addition to severity of the disease, cancer patients developed COVID-19 severe symptoms more rapidly than those without cancer (median time to severe events: 13 days *vs.* 43 days)^1^. Furthermore, cancer patients with COVID-19 had more prolonged hospital stays. In a retrospective study, 35.7% of COVID-19 cancer patients were discharged after 13.5 days, while 35.7% remained inpatient after 19 days^5^. In contrast, a meta-analysis showed that the average discharge rate of non-cancer COVID-19 patients was 52% during a 38-day period^16^. Together, these data suggest that cancer patients may have an increased risk of COVID-19 and a poorer prognosis.

There are limitations in these cohort studies. They were not prospectively randomized and had a relatively small sample size. Each cohort included diverse cancer types and tumor stages as well as treatment modalities. Furthermore, as treatment strategies for COVID-19 infection continue to improve, older reports may no longer be relevant.

One study indicated that the percentages of SARS-CoV-2 infections, severe events, and deaths in cancer patients undergoing usual SARS-CoV-2 treatment were not higher compared to the general population, due to a low percentage of treatment-related adverse events (5.5%)^17^. Similarly, breast cancer patients did not appear to have a higher risk of severe COVID-19 than the general population^18^. Thus, more rigorously designed studies are needed to confirm whether cancer patients develop more severe COVID-19 symptoms upon infection with SARS-CoV-2.

## Clinical characteristics of cancer patients with COVID-19

For cancer patients with COVID-19, a retrospective study showed that the most common symptoms at presentation were fever, dry cough, and fatigue^5^. Although the non-cancer and cancer patients with COVID-19 have similar clinical presentation, fatigue and dyspnea symptoms appear to be more frequent among the latter^5,6,19^. Especially among COVID-19 patients with lung cancer or lung metastasis, dyspnea was found to occur much earlier from the onset of COVID-19 diagnosis, when compared to both the non-cancer patients and those with other cancer types^5,6^. This was probably because patients with lung cancer or lung metastasis have a worse baseline lung function and endurance, and thus are more likely to develop severe anoxia and COVID-19 progression.

Besides respiratory symptoms, cancer patients with COVID-19 may also develop a variety of complications^1,20–22^. The most common complications and cause of deaths in these patients were acute respiratory distress syndrome (ARDS) (28.6%), followed by pulmonary embolism (7.1%), septic shock (3.6%), and acute myocardial infarction (AMI) (3.6%)^5^. However, another study showed that common complications were liver injury (36.5%), ARDS (17.3%), sepsis (15.4%), myocardial injury (15.4%), renal insufficiency (7.7%), and multiple organ dysfunction syndrome (5.8%) in 52 cancer patients with COVID-19^19^. Given that cancer patients without COVID-19 also have a high risk of these complications, attributing these symptoms in COVID-19 cancer patients solely to underlying cancer is difficult.

## Radiographical and laboratory findings of cancer patients with COVID-19

For both general and cancer patients with COVID-19, the most frequent feature in chest CT imaging was ground-glass opacity, and the second was patchy consolidation^5,23^. Notably, air bronchogram and interstitial abnormal findings were common in general COVID-19 patients, but not in cancer patients^5,23^. COVID-19 cancer patients had a higher percentage of bilateral lung involvement than regular patients^3,5^.

Besides radiographical findings, there are biochemical features associated with COVID-19^24,25^. One study showed the cytokine release syndrome may be a sign of disease progression^26^. Higher levels of IL-6 and IL-10 as well as lower levels of CD4+ and CD8+ T cells found in COVID-19 patients correlated with the severity of the disease^26^. Compared with the general COVID-19 patients, cancer patients with COVID-19 had similar blood counts^3,5,19,20^. However, there was a higher percentage of COVID-19 cancer patients presenting with anemia^4,5^. It is possible that anemia in COVID-19 cancer patients is a consequence of nutritional deficiency and an immunosuppressive state, leading to increased susceptibility to respiratory pathogens. A comparison of clinical features in general and cancer COVID-19 patients is shown in **Table 1**.

## Treatment of COVID-19 in cancer patients

### Oxygen therapy for COVID-19 patients

The most important symptomatic treatment for COVID-19 patients is oxygen therapy^27^. For cancer patients with COVID-19, there was a higher percentage of patients who received oxygen therapy^5^. The higher proportion of COVID-19 patients with cancer requiring oxygen therapy and mechanical ventilation may be related to more severe disease and an immunosuppressive state in cancer patients, who are more susceptible to secondary lung infection with other pathogens.

### Antiviral treatment for COVID-19 patients

Currently, there is no antiviral drug that is specifically effective against SARS-CoV-2. Several clinical studies have indicated that remdsivir, arbidol, and chloroquine may have moderate benefits for treating COVID-19^24,28–30^. Larger clinical studies need to confirm these results. For cancer patients with COVID-19, the use of antiviral drugs did not yield any different outcomes compared with general COVID-19 patients. About 71.4% of cancer patients with COVID-19 received at least one antiviral agent, including arbidol, lopinavir/ritonavir, ganciclovir, and ribavirin, while 32.1% received two or more antiviral agents^5,6^.

### Immune enhancement therapy for COVID-19 patients

Given that COVID-19 cancer patients may have systemic immunosuppression, intravenous immunoglobulin may be a promising treatment of COVID-19. One study showed that 12 out of 28 cancer patients with COVID-19 received intravenous immunoglobulin treatment. However, the study could not provide adequate information about efficacy due to the limited sample size and lack of a randomized control group^5^.

### Anti-inflammatory therapy for COVID-19 patients

The rationale for anti-inflammatory therapy is based on the premise that COVID-19 induces a cytokine storm with deleterious effects on tissues^31^. In a controlled, open-label trial, the use of dexamethasone in hospitalized patients with COVID-19 resulted in lower 28-day mortality among those receiving either invasive mechanical ventilation or oxygen alone^32^. For cancer patients with COVID-19, the use of systemic corticosteroids remains controversial. Given that cancer patients are already at a higher risk of opportunistic infections, the use of corticosteroids may not be effective in mitigating COVID-19 symptoms. Indeed, one study showed that corticosteroids did not reduce the incidence of severe events in cancer patients with COVID-19^5^. Blood purification therapy is an alternative treatment to reduce cytokine storms and benefit critically ill COVID-19 patients. One report showed that the therapy was effective in managing cytokine storms and pathogenic antibodies in three critically ill COVID-19 patients with profound inflammations^33^. However, larger randomized data were lacking. Furthermore, multi-disciplinary efforts are needed to achieve increased availability of blood purification therapy for COVID-19 cancer patients.

### Convalescent plasma therapy for COVID-19 patients

Convalescent plasma therapy has also been explored to alleviate COVID-19 symptoms^25,31^. Of note, there are some potential risks and ethical issues associated with its usage, including thrombotic risk and the selection of donors. Given that cancer patients with COVID-19 may have a more rapid disease progression, convalescent plasma therapy may be particularly beneficial in this population. To date, there is no report about the effectiveness of convalescent plasma therapy in this patient population. Current reported studies on treatments for general and cancer COVID-19 patients are summarized in **Table 2**.

## Therapies for COVID-19 associated with anti-tumor therapies

It is known that immune tolerance is a key part of tumorigenesis and anti-tumor therapy resistance^36^. Similar to cancer therapy, one method of vaccine development may be a T cell epitope vaccine to enhance the T cell recognition of virus-infected cells. The regimen used to prevent or reduce the cytokine storm in cancer patients during CAR-T cell therapy may also be used to reduce the risk of cytokine storm in COVID-19 patients^36^. It is known that IL-6 is a critical cytokine involved in cancer and inflammation. High levels of IL-6 predict poor prognoses of patients with COVID-19^37^. Tocilizumab, an IL-6 receptor-targeted antibody, is approved to alleviate CAR-T cell-induced cytokine release syndrome (CRS) in cancer patients^34^. Among 129 patients hospitalized for COVID-19, those who received tocilizumab in addition to standard treatment were significantly less likely to need ventilation or die within 2 weeks, when compared with those who received standard treatment alone^34^. Therefore, antibodies targeting the IL-6 receptor (tocilizumab and sarilumab), IL-6 (siltuximab), and other receptor antagonists (α1-adrenergic receptor antagonist, prazosin) for mitigating cytokine storm are promising therapeutic strategies for the treatment of cancer patients with COVID-19^35^.

Besides IL-6, other cytokines such as type-I interferon, IL-1β, IL-7, IL-17, and TNF-α are central to the pathophysiology of COVID-19^38^ as well as to cancer pathogenesis and the therapeutic response^39^. In particular, IL-17 is a critical cytokine associated with immune responses in both cancer and COVID-19 patients^40^. Given that anti-IL-17 antibodies have demonstrated a therapeutic role in the treatment of cancer and lung infection by H1N1 and AIDS^41^, this approach might be useful to control COVID-19 in cancer patients.

## The influence of COVID-19 on cancer diagnosis and management

COVID-19 is closely associated with an inflammatory outburst, oxidative stress, and other pathophysiological abnormalities, which can exert tremendous impact on the assessment of cancer and treatment options^20,42–44^. Notably, a retrospective study showed that there were significant increases in levels of several serum cancer biomarkers in mild cases of COVID-19, when compared to normal control subjects. These cancer biomarkers were further increased in severe cases of COVID-19^42^. These alterations may affect the positive and negative predictive values of several tumor-related biomarkers, thereby making it challenging to accurately assess and determine cancer diagnosis, disease progression, and therapeutic options.

To reduce the risk of infection in cancer patients, several noncontact diagnostic and screening resources may be used. For example, screening colonoscopy may be delayed or canceled during the pandemic, in preference for DNA-based stool sample tests for colorectal cancer screening^45^. For cancer patients, more vigorous personal protection and monitoring should be encouraged^46,47^. Decisions regarding surgical intervention during this time must undergo rigorous ethical and clinical evaluations. Except in emergency cases, it is better to use multidisciplinary conferences to gather consensus regarding surgical therapy because of the higher risk of SARS-CoV-2 infection^6,48^.

Although patients undergoing chemotherapy seemed to be at a higher risk of severe illness from COVID-19^6,46^, delaying chemotherapy is not recommended, because cancer progression may be exacerbated by COVID-19-elicited inflammatory signals. Therefore, dose reduction of chemotherapy could be considered. Surprisingly, cancer patients undergoing radiotherapy did not show a higher risk of having any severe events from COVID-19^6^, which may be attributed to the activation of the immune system by radiotherapy^49^. Nevertheless, radiotherapy may need to be safely delivered in a hypofractionated fashion where feasible, to minimize the number of visits to treatment centers^50^. Regarding targeted therapy, patients who develop fever should undergo COVID-19 testing before continuing treatment^51^.

Extensive preclinical and clinical data exist on the safety and efficacy of immunotherapy in cancer patients with viral infections^52^. However, the impact of immune checkpoint inhibitors (ICIs) on COVID-19 pathogenesis and their safety during acute COVID-19 infection is largely unknown. It may be difficult to distinguish features of COVID-19 infection from immune-mediated toxicities related to ICIs^53^. For example, it is challenging to distinguish anti-PD-1-induced pneumonitis from SARS-CoV-2 infection, because the clinical features are similar. This similarity may also complicate the ensuing management of patients receiving anti-PD-1 therapy with pneumonitis. Furthermore, patients with severe COVID-19 are more likely to have CRS^20,53^ and overactivated T cells^54^, which is also a phenomenon of immune hyperactivation typically described in the setting of T cell-engaging immunotherapy including CAR-T cell therapy and ICIs^53^. CRS and overactivated T cells can cause severe immune-mediated injuries to many organs^55^ and thus may increase the risk of severe events in cancer patients with COVID-19. Notably, one study showed that prior anti-PD-1 therapy in lung cancer was not associated with an increased risk of severity of COVID-19^56^. How SARS-CoV-2 virus infection impacts the effectiveness and outcome of anti-cancer therapy largely depends on our ongoing understanding of biochemical and pathophysiological mechanisms of COVID-19.

## Conclusions

COVID-19 infection has a tremendous impact on cancer diagnosis, prognosis, and therapeutic effects. Emerging studies show a worse trend among cancer COVID-19 patients compared to non-cancer COVID-19 patients. However, other studies also indicate that the percentages of SARS-CoV-2 infection and severe events in cancer patients are not higher compared to the general population. Therefore, ongoing investigation is needed to better understand the comorbidity of COVID-19 and cancer. The efficacy and safety of COVID-19 treatment approaches in cancer patients also requires further investigation. Because the majority of the reports associated with cancer patients with COVID-19 involve cohort studies with a relatively small sample size, limited clinical information, high heterogeneity of tumor stages and cancer types as well as diverse treatments, current understanding of the comorbidities are limited. Clinical outcomes and the biological basis for the comorbidity of cancer and COVID-19 remain to be resolved. The possibility of COVID-19 recurrence brings a new sense of urgency to develop strategies to optimize the management and care of cancer patients with COVID-19. Although we intend to give a timely summary of the published studies on the impact of COVID-19 on cancer patients, our conclusions could become outdated as studies on the topic are expected to be reported during the ongoing pandemic crisis. We hope to bring attention to the symptoms, prognoses, and treatment options of cancer patients with COVID-19, and to stimulate research into the daunting challenge of addressing COVID-19 and cancer comorbidities.

## Figures and Tables

**Table 1:** Clinical features in non-cancer and cancer patients with COVID-19

Clinical features		Non-cancer patients with COVID-19	Cancer patients with COVID-19
Clinical charac teristics	Similar:	Fever (88.7%)	Fever (78% to 82.1%)
		Cough (67.8%)	Dry cough (74% to 81%)
		Nausea or vomiting (5%)	Nausea or vomiting (5.71%)
		Diarrhea (3.8%)^3–5^	Diarrhea (12%)^5,6,14^
	Different:	Fatigue (38.1%)	Fatigue (64.3%)
		Dyspnea (21.9%)	Dyspnea (50%) tachypnea (14.3%)
		From onset to dyspnea was 8 days^3–5,16^	From onset to dyspnea was 1 or 5 days for lung cancer or other cancer patients, respectively^5,6^
Radiographical findings (Chest CT imaging)	Similar:	Ground-glass opacity (65%)	Ground-glass opacity (69% to 75%)
		Patchy consolidations (50%)^3,24^	Patchy consolidation (46.3%)^5,14^
	Different:	Air bronchogram (47%) Interlobular septal thickening (35%) Adjacent pleura thickening (32%)	Interstitial abnormal findings including reticular appearance, fibrous trips and interlobular septal thickening (14.3%)
		Bilateral lung involvement (51.8%)^3,24^	Bilateral lung involvement (78.6% to 91%)^5,14^
Laboratory find ings	Similar:	Lymphopenia (83.2%)	Lymphopenia (82.1%)
		Leukopenia (33.7%)	Leukopenia (32.1%)
		Elevated CRP (86%)	Elevated CRP (82.1%)
		Elevated myoglobin (15%)	Elevated serum globulin (39.3%)
		Elevated D-Dimer (36%)	Elevated D-Dimer (39.3%)
		Low level of serum albumin (98%)^3,4,20^	Low level of serum albumin (89.3%)^5,19^
	Different:	Anemia (51%)	Anemia (75%)
		Elevated LDH (76%)	Elevated LDH (50%)
		Elevated ESR (85%)^3,4,20^	Elevated ESR (57.1%)^5,19^

CRP, C-reactive protein; LDH, lactate dehydrogenase; ESR, erythrocyte sedimentation rate.

**Table 2:** COVID-19 treatments for non-cancer and cancer patients with COVID-19

Treatment	Non-cancer patients with COVID-19	Cancer patients with COVID-19
Oxygen therapy	Oxygen support	Oxygen support
	High-flow nasal cannula	Invasive mechanical ventilation
	Prone position ventilation	Endotracheal intubation and invasive ventilation
	Extracorporeal membrane oxygenation^24,27^	Extracorporeal membrane oxygenation^5,6,14^
Antiviral therapy	Remdesivir	Oseltamivir
	Arbidol	Umifenovir
	Chloroquine	Arbidol
	Lopinavir/ritonavir	Lopinavir/ritonavir
	Nucleoside analogues	Ganciclovir
	Neuraminidase inhibitors^24,25,28–30^	Ribavirin^5,6,14^
Immune enhancement therapy	Interferon	Interferon
	Intravenous immunoglobulin	Intravenous immunoglobulin^5,14^
	Thymosin alpha-1^25,31^	
Anti-inflammatory therapy	Corticosteroid	Systemic corticosteroid
	Methylprednisolone	Tocilizumab^5,6,14,34^
	Dexamethasone	
	Blood purification therapy	
	Tocilizumab and sariluma	
	Siltuximab	
	Prazosin^31–35^	
Convalescent plasma therapy	Convalescent plasma therapy	No relative reports
	Plasma globulin specific to SARS-CoV-2^25,31^	The safety and the efficacy need further evaluation
